# Heart-Cutting Bidimensional Liquid Chromatography for the Simultaneous Analysis of Veterinary Drugs Residues and Nucleotide Monophosphates in Sheep’s Milk

**DOI:** 10.3390/foods13060872

**Published:** 2024-03-13

**Authors:** Noelia Caballero-Casero, Diego García-Gómez, José Luis Pérez Pavón, Encarnación Rodríguez-Gonzalo

**Affiliations:** 1 Analytical Chemistry, Nutrition and Food Science Department, University of Salamanca, Plaza de Los Caídos s/n, 37008 Salamanca, Spain; a42caasn@uco.es (N.C.-C.); jlpp@usal.es (J.L.P.P.); erg@usal.es (E.R.-G.); 2 Department of Analytical Chemistry, Institute of Chemistry for Energy and Environment, University of Córdoba, Marie Curie Annex Building, Campus de Rabanales, 14071 Cordova, Spain

**Keywords:** two-dimensional liquid chromatography, heart-cutting 2D-LC, veterinary drug residue, nucleotide monophosphates, sheep’s milk

## Abstract

Sheep’s milk is a significant source of nucleotide monophosphates (NMPs) but can also contain undesirable residues from veterinary drugs, posing a potential human health risk. This study introduces a novel application of two-dimensional liquid chromatography (2D-LC), in heart-cutting mode, for the simultaneous determination of nucleotides and veterinary drug residues in sheep’s milk. 2D-LC allows for the separation of these compounds in a single chromatographic run despite their differing physicochemical properties. The proposed method separates six veterinary drug residues and five NMPs in a single injection. The compounds were separated using a C18 reversed-phase column in the first dimension and a Primesep SB analytical column in the second dimension. The method performance was evaluated in terms of linearity range, detection and quantification limits, matrix effects, precision, and accuracy. The results demonstrated good linearity and sensitivity, with quantification limits allowing for the quantification of veterinary drugs at the maximum residue level and nucleotides at typical levels found in milk samples. The method has been successfully applied to the analysis of sheep’s milk samples acquired from local supermarkets, with recoveries within a range of 70–119% and 82–117% for veterinary residues and NMPs, respectively.

## 1. Introduction

Milk and its derivatives are fundamental foods in the human diet, especially due to their contribution of high-quality proteins and other essential nutrients, such as nucleotides. Nucleotides are organic compounds that are part of nucleic acids and participate in numerous metabolic and physiological processes, such as protein synthesis, regulation of gene expression, cellular signaling, and the immune system [1]. Sheep’s milk is an important source of nucleotides as it contains a higher concentration of these compounds compared to cow’s or goat’s milk [2].

However, milk can also contain undesirable residues from the use of veterinary drugs for the treatment or prevention of diseases in the producing animals. These residues can pose a risk to human health, causing allergic reactions, bacterial resistance, or toxic effects [3]. Therefore, the European Union has established maximum residue limits (MRLs) for different pharmacologically active substances in animal-derived products, including sheep’s milk [4]. Among these substances are antibiotics, which are widely used in sheep farming to combat bacterial infections. Some commonly used antibiotics include trimethoprim (MRL 50 ng/mL) and fluoroquinolones (such as enrofloxacin and ciprofloxacin, MRL 100 ng/mL), which act by inhibiting the synthesis of folic acid and bacterial DNA, respectively [5]. Another group of drugs used in sheep farming are anthelmintics, such as albendazole (MRL 100 ng/mL), which inhibit the growth and reproduction of intestinal parasites [6]. Albendazole is metabolized in the animal’s body, giving rise to several metabolites, such as albendazole sulfoxide, albendazole sulfone, and albendazole-2-amino sulfone, which can persist in milk [7]. The hitherto unexplored joint determination of these compounds (i.e., nucleotides and veterinary drugs) in a single analysis would allow for a simpler more cost-effective quality control of sheep’s milk.

However, the simultaneous determination of nucleotides and veterinary residues in milk poses an analytical challenge due to the differences in the physicochemical properties of these compounds. Nucleotides are polar and ionic molecules that require hydrophilic and ionic chromatographic conditions for their separation, while veterinary residues are apolar or moderately polar molecules that require hydrophobic chromatographic conditions for their retention [8]. Therefore, the use of different analytical techniques for each group of analytes becomes necessary, which implies greater complexity, time, and cost of the analysis.

In this context, two-dimensional liquid chromatography (2D-LC) emerges as a powerful tool to solve complex analytical problems by combining two different chromatographic systems in a single instrumental platform [9]. 2D-LC offers higher separation and resolution capabilities than one-dimensional liquid chromatography by taking advantage of complementary interactions between analytes and stationary phases in each dimension [10]. Furthermore, 2D-LC allows for the reduction of analysis time and solvent consumption by using shorter columns and faster gradients [11].

The aim of this study is to develop and validate an analytical method based on 2D-LC for the simultaneous determination of nucleotides and veterinary residues in sheep’s milk in a single injection. To the best of our knowledge, this is the first study to achieve the chromatographic separation of both groups of analytes simultaneously by applying this technique to this type of matrix. For this purpose, the separation of six veterinary drug residues (trimethoprim, albendazole sulfoxide, albendazole sulfone, albendazole-2-amino sulfone, enrofloxacin, and ciprofloxacin) in the first dimension, based on hydrophobic interactions, and five NMPs (cytidine, uridine, inosine, guanosine, and adenine monophosphate) in the second dimension, based on ionic interactions, has been optimized. The performance of the method has been evaluated in terms of selectivity, linearity, precision, accuracy, limit of detection, and limit of quantification. Additionally, the method has been applied to real sheep’s milk samples.

## 2. Material and Methods

### 2.1. Sample Collection

Sheep’s milk samples were acquired from local supermarkets in Salamanca (Spain). UHT milk tetra brick containers of 1 L from the principal producer brand were purchased and kept at 4 °C and dark conditions until their analysis. The whole content of commercial packages was homogenized by shaking prior to analysis. Once opened, milk was analyzed within the following week.

### 2.2. Chemicals and Apparatus

All chemicals were of analytical grade and used as supplied by manufacturers. Analytical standards of the veterinary drugs trimethoprim (TMP), albendazole 2-amino sulfone (ANH_2_), enrofloxacin (ERF), ciprofloxacin (CPF) and the NMPs cytidine 5′-monophosphate disodium salt (CMP), adenosine 5′-monophosphate disodium salt (AMP), inosine 5′-monophosphate disodium salt (IMP), uridine 5′-monophosphate disodium salt (UMP), and guanosine 5′-monophosphate disodium salt (GMP) were purchased from Sigma-Aldrich (Steinheim, Germany). Dr Ehrenstorfer (Augsburg, Germany) supplied the standards of the veterinary drugs albendazole sulfoxide (ASO) and albendazole sulfone (ASO_2_). Acetonitrile LC-grade was obtained from Merck (Darmstadt, Germany). Hydrochloric acid (HCl, 37% *w*/*w*), acetic acid and formic acid were supplied by Scharlau (Barcelona, Spain). Salts used as mobile phase modifiers, ammonium acetate and sodium acetate, were purchased from Sigma-Aldrich (Steinheim, Germany). Deionized water with 18.2 mΩ cm was obtained from a Wasserlab Ultramatic water purification system (Noain, Spain).

Individual stock solutions of TMP, ERF, ASO, ANH_2_, and ASO_2_ were prepared by dissolving them in acetonitrile (500 mg L^−1^). To enhance the solubility of ASO, ANH_2_, and ASO_2_, HCl at 0.1% was added. Individual stock solutions of CPF, CMP, UMP, GMP, IMP, and AMP (500 mg L^−1^) were prepared in deionized water. Mixed standard solutions of veterinary drugs (CPF, TMP, ERF, ASO, ANH_2_, and ASO_2_) and of NMPs (CMP, UMP, GMP, IMP, and AMP) were prepared by appropriate dilution with acetonitrile/water (50/50%, *v*/*v*) and water, respectively. Working solutions were prepared on a weekly basis by dilution and stored at 4 °C in darkness.

For sample treatment, the following equipment was utilized: a pH meter from Metrohm (Herisau, Switzerland), a centrifuge MPW-350R from MPW Medical Instruments (Warsaw, Poland), and a vortex-shaker from Reax Heidolph (Schwabach, Germany).

### 2.3. Centrifugation-Assisted Ultrafiltration (CUF) of Milk Samples

The milk samples underwent a centrifugation-assisted ultrafiltration (CUF) process. For this purpose, disposable Corning TM Spin-XR UF 20 centrifuge tubes with a 5 kDa molecular weight cutoff (MWCO) were used. These devices are equipped with a vertical polyethersulfone (PES) membrane and a fine-channel filtration chamber, which reduces membrane saturation and enables rapid filtration. A total of 10 mL milk samples were centrifuged for 20 min at 3000 rpm. The milk extract without high molecular weight compounds was collected and submitted to the liquid chromatography-diode array analysis.

### 2.4. Two-Dimensional Chromatographic Separation (2D-LC) and Ultraviolet Detection (DAD)

2D-LC in heart-cutting mode was selected for the simultaneous analysis of both groups of compounds. The chromatographic separation of the analytes was achieved in a 1290 Infinity 2D-LC HPLC system from Agilent (Waldbronn, Germany) equipped with two binary pumps, an autosampler, an 8-port 2-position valve with a 100 µL transfer loop and two Diode-Array Detectors (DAD). A C18 reversed-phase column ACE 3 C18-PFP (100 mm × 2.1 mm, particle size 3 µm) thermostated at 35 °C was used for the separation of the veterinary drugs performed at the first LC dimension (1D). For the separation of the NMPs at the second LC dimension (2D), a Primesep SB analytical column (100 mm × 2.1 mm, particle size 5 µm) was selected.

Chromatographic runs were simultaneously obtained for veterinary drugs (1D) and NMPs (2D). The mobile phase used in the first-dimensional separation consisted of 0.1% (*v*/*v*) formic acid (pH: 3) as solvent A and acetonitrile as solvent B, with the following linear gradient: from 90% to 85% of A for 5 min, then it decreases up to 80% in 0.5 min and kept constant for 2.5 min; next, from 80% to 10% of A for 2 min, and finally, after another 2 min, the column was equilibrated at the initial mobile phase composition for 7.5 min. The injection volume was 50 µL and the flow rate was set at 0.2 mL min^−1^. As the heart-cutting mode was used, at 1.6 min of the 1D, a sample fraction was accumulated in the sample loop for 0.2 min and transferred to 2D by a six-port valve. For the 2D-LC, the mobile phase consisted of a mixture of acetonitrile:water (10:90%, *v*:*v*) containing sodium acetate (60 mM) and adjusted with acetic acid to pH 4.5, in isocratic mode.

The veterinary drugs were determined at 285 nm while the NMPs were determined at 270 nm. Quantification was carried out by external calibration with standards in acetonitrile/water (50/50%, *v*/*v*).

### 2.5. 2D-LC-DAD Method Performance

The analytical performance of the method was evaluated in terms of linearity range, method detection and quantification limits, matrix effects, precision, and trueness. For this purpose, the criteria outlined in the European Commission decision 2002/657/EC [12] were followed. This guideline defines acceptable performance criteria to assess the suitability of an analytical method for its intended purpose.

## 3. Results and Discussion

### 3.1. 2D-LC-DAD Method Development

Two-dimensional liquid chromatography is an analytical methodology that couples two distinct chromatographic modes, substantially increasing separation efficiency. The combination of different chromatographic mechanisms facilitates the separation of mixtures of compounds that are difficult to separate when using a single chromatographic mode in a conventional liquid chromatograph. In this study, heart-cutting mode has been used, which consists of the transfer of an eluent fraction between the first and second chromatographic columns.

Therefore, firstly, the chromatographic separation of veterinary residues in 1D was optimized. For this purpose, different elution programs were tested, and all the details about the elution programs are given in Table S1. Hydrophobic interactions between the stationary phase and the analytes, which display moderate polarity (Table S2), are favored by increasing the percentage of aqueous solvents in the mobile phase. Therefore, in isocratic mode, the composition of the mobile phase was studied between 98% and 50% of solvent B, although it was not possible to achieve a baseline separation of the chromatographic peaks. Therefore, a gradient elution mode was preferred. The selection of the mobile phase gradient was based on the total time of analysis and the chromatographic factors, which are listed in Table 1. Additionally, during the optimization of the mobile phase composition on 1D, it was considered that all the NMPs studied elute in a single fraction equivalent to 40 µL, for a flow rate of 0.2 mL/min, for their posterior separation in 2D. The final chromatographic program provided separation factors > 1.1 (Table 1) and a total run time of 20 min.

**Figure 1:**
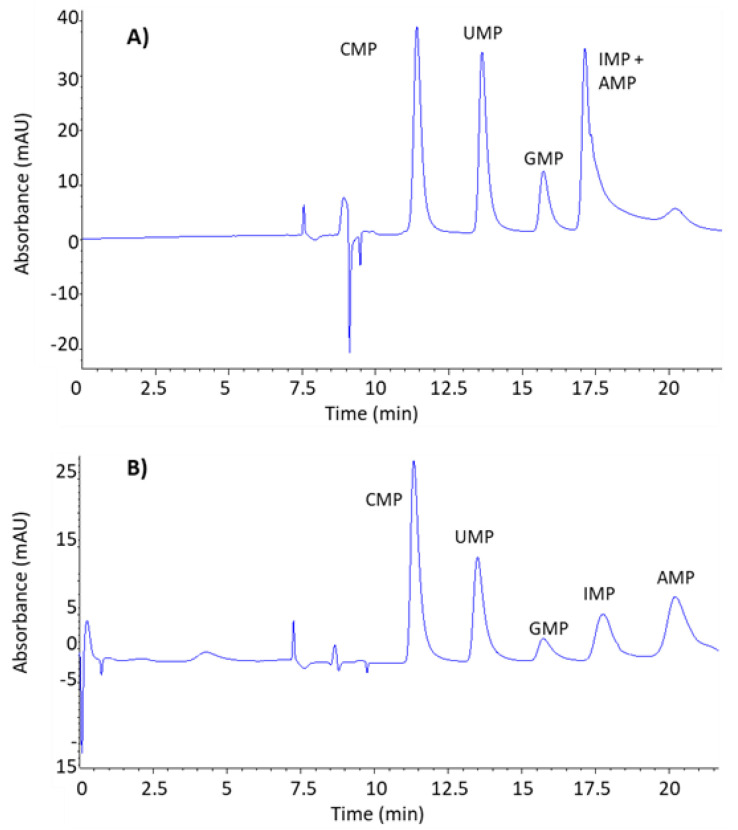
Chromatograms of NMPs acquired in LC-DAD at 270 nm under the same chromatographic conditions but different mobile phase compositions: (**A**) H_2_O/Acetonitrile (98/2%, *v*/*v*) + 30 mM ammonium acetate as solvent A, and H_2_O/Acetonitrile (98/10%, *v*/*v*) + 60 mM ammonium acetate as solvent B, both at pH: 4.5. And (**B**) H_2_O/Acetonitrile (98/2%, *v*/*v*) + 30 mM sodium acetate, pH: 4.5, as solvent A; and H_2_O/Acetonitrile (98/10%, *v*/*v*) + 60 mM sodium acetate, pH: 4.5, as solvent B.

Once the chromatographic conditions of 1D were optimized, it was necessary to study the optimal conditions for the chromatographic separation of the NMPs. The stationary phase used for the separation of NMPs was a reversed-phase with strong embedded basic ion-pairing groups. It contains a C18 carbon chain and basic quaternary amine groups. The selection of this stationary phase was based on the fact that it offers both hydrophobic and ion-pairing interactions. The C18 carbon facilitates hydrophobic interactions with the nucleotide bases, which are influenced by their hydrophobicity. The basic quaternary amine groups embedded in the column can act as ion-exchange sites, interacting with the negatively charged phosphate groups of the nucleotide monophosphates. This ion-exchange mechanism can play a significant role in the retention and separation of nucleotides, depending on the pH and ionic strength of the mobile phase. Thus, two different mobile phase modifiers were tested, ammonium acetate, previously used for the separation of NMPs, and sodium acetate, a greener modifier. The same elution program was applied, a linear gradient from 100% solvent A to 0% in 12 min, 4 min hold, and return to initial conditions in 4 min. As it is shown in Figure 1A, the mobile phase containing ammonium acetate (Solvent A: H_2_O/Acetonitrile (98/2%, *v*/*v*) + 30 mM ammonium acetate. Solvent B: H_2_O/Acetonitrile (98/10%, *v*/*v*) + 60 mM ammonium acetate) and adjusted to pH 4.5 was not able to achieve baseline separation for all the analytes. The mobile phase containing sodium acetate (Solvent A: H_2_O/Acetonitrile (98/2%, *v*/*v*) + 30 mM sodium acetate, 4.5 pH. Solvent B: H_2_O/Acetonitrile (98/10%, *v*/*v*) + 60 mM sodium acetate, 4.5 pH) provided stronger retention of the analytes leading to the chromatographic resolution of all analytes, although the peaks obtained were too wide (Figure 1B).

In order to obtain narrower chromatographic peaks for NMPs, the elution program was optimized (Table S3). Despite different gradient elution methods being tested, the best results in terms of peak shapes were achieved with an isocratic method. Thus, a mixture of acetonitrile:water (10:90%, *v*:*v*) containing sodium acetate (60 mM) and adjusted with acetic acid to pH 4.5, in isocratic mode, was selected as optimal for the separation of NMPs. The chromatographic factors are listed in Table 2.

### 3.2. Sample Treatment

Sheep’s milk contains a higher fat content (6.4%) than cow’s (3.5%) or goat’s (4.0%) milk. In order to eliminate the lipids, the use of Captiva EMR-Lipid cartridges was tested. The procedure is very simple and is based on a filtration that removes lipids and some proteins through a combination of size exclusion and hydrophobic interactions between the sorbent and the aliphatic chains. However, the high content of fats and proteins in sheep’s milk led to the occlusion of the cartridges, making necessary a first step of protein elimination. For this reason, the use of Captiva was discarded.

A second approach based on protein and macromolecule removal by ultrafiltration was studied. The centrifugal ultrafiltration devices (disposable Corning TM Spin-XR UF devices for 20 mL samples) used had a molecular weight cut-off (MWCO) of 5 kDa. Milk samples were introduced in the filtration chamber of tubes, which are equipped with a vertical polyethersulfone membrane and a narrow channel, which reduces membrane saturation and allows for rapid filtration. After 20 min of centrifugation at 3000 rpm, the whole aliquot sample was filtered and macromolecules removed, obtaining a clear-yellow extract that could be directly analyzed by 2D-LC-DAD.

### 3.3. 2D-LC-DAD Analytical Performance

The analytical method developed for the determination of veterinary residues and NMPs in milk has been evaluated in terms of linearity, sensitivity, selectivity, recovery, and precision. The evaluation of these parameters was carried out based on the European Commission decision 2002/657/EC [12], which sets the performance criteria for analytical methods for the determination of organic residues or contaminants in foodstuffs of animal origin.

#### 3.3.1. Linearity and Sensitivity

Table 3 presents the calibration parameters, and the method quantitation limits (MQLs) established for the analysis of veterinary drugs in 1D and NMPs in 2D. Calibration curves were obtained by plotting the area of nine standard calibration solutions versus concentration, with the highest tested concentration being 0.5 mg L^−1^ and 100 mg L^−1^ for veterinary drugs and NMPs, respectively. Correlation between peak areas and analyte concentrations was determined by linear regression and obtaining correlation coefficients above 0.97 in all the cases, indicating a good fit. Linearity was confirmed through residual plots against analyte concentration, showing a random distribution of residuals within a horizontal band and a random series of positive and negative residuals.

Methods quantification limits (MQLs) were calculated from six non-fortified milk samples submitted to the whole proposed method, by using a signal-to-noise ratio of 10, determined by the ratio of peak height for each target analyte to the peak height of noise; resulting in a range of 0.01–0.025 mg L^−1^ for veterinary drugs and 0.25–0.75 mg L^−1^ for NMPs. This range enables the quantification of veterinary drugs at the maximum residue level (MRL) (0.05–0.1 mg L^−1^ [4]) and NMPs at the typical levels found in milk samples.

#### 3.3.2. Selectivity

The potential interferences due to the coelution of matrix compounds with the analytes were evaluated through the visual inspection of six unfortified milk samples. In the case of veterinary residues, no chromatographic peaks were detected at the retention times of the compounds of interest. In the case of the NMPs, which are present in the milk samples, selectivity was studied through the absorbance spectrum at the retention times of the analytes. The spectra were identical to those obtained in the standard solutions. Therefore, it was assumed that the determination of the analytes was not affected by matrix components, thus external calibration for the quantification of the analytes can be performed.

#### 3.3.3. Recovery

To the best of our knowledge, certified reference materials for veterinary drug residues or NMPs in sheep’s milk are not available. Thus, the extraction efficiency of the developed method was evaluated by the analysis of fortified milk samples. Following the European Commission decision 2002/657/EC [12], milk samples (*n* = 3) were fortified at three different concentration levels. In the case of veterinary residues, they were fortified at 0.5, 1, and 1.5 times the MRL [4], while the NMPs were fortified at levels of 5, 10, and 15 mg L^−1^.

The recoveries for veterinary residues (Table 3) at the three levels of fortification were in the range of 70–108%; 78–120%; 84–114%, respectively, with standard deviations between 1 and 10%. Recoveries obtained for NMPs, at the three levels of concentration studied were in the range of 80–85%; 83–119%; 85–118%, respectively, with standard deviations below 14% in all the cases. These results agreed with the 2002/657/EC decision which considers that recoveries for analyte concentrations below 0.01 mg L^−1^ should be in the interval 70–130%, while for concentrations above 0.01 mg L^−1^ between 80–120%.

#### 3.3.4. Precision

Precision was studied in terms of repeatability and within-laboratory reproducibility and expressed as the percentage of coefficient of variation (CV%). Eighteen aliquots of milk samples (*n* = 18), fortified at three different concentration levels were analyzed over three days (six aliquots per day), utilizing freshly prepared mobile phases and standard solutions. In the case of veterinary residues, they were fortified at 0.5, 1, and 1.5 times the MRL [4], while the NMPs were fortified at levels of 5, 10, and 15 mg L^−1^. The repeatability was calculated as the square root of the average intra-day variances obtained. The intra-laboratory reproducibility was calculated as the square root of the sum of the mean intra-day variance and the inter-day variance. The obtained CV (%) under repeatability and reproducibility conditions (Table 3) varied within 3–9%, 2–5%, and 1–6% intervals for veterinary residues; and 3–12%, 4–10%, and 3–8% for NMPs. These results align with the 2002/657/EC Decision, stipulating that the CV (%) for intra-laboratory reproducibility conditions should not exceed 15%.

### 3.4. Analysis of Sheep’s Milk Samples

**Figure 2:**
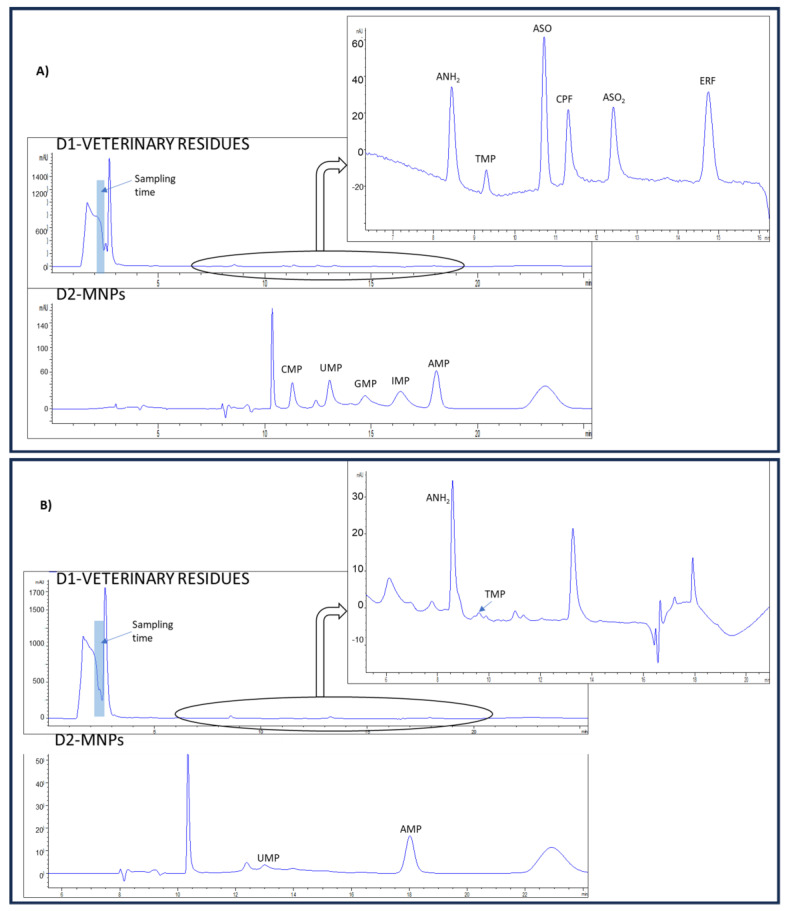
Chromatograms were obtained from (**A**) sheep’s milk sample fortified at MRL of veterinary drug residues and NMPs at 15 mg L^−1^ and (**B**) non-fortified sheep’s milk sample.

To evaluate the appropriateness of the proposed method for routine control of the veterinary drug residue and NMP content, three sheep’s milk tetra-bricks from two different brands were analyzed. Table 4 shows the concentration found in the analyzed samples of the analytes, as well as the recoveries calculated for the sheep’s milk samples fortified at the MRL of veterinary drugs and 15 mg L^−1^ NMPs. The recovery values obtained were within a range of 70–119% and 82–117% for veterinary residues and NMPs, respectively. No interference from matrix components was observed in any of the analyzed samples. As can be observed, the veterinary residue ANH_2_ was quantified at a concentration above the MRLs in samples 1 and 2. This highlights the importance of the determination of these compounds to ensure food security. Chromatograms for a milk sample, non-fortified and fortified at MRL of veterinary drugs and 15 mg L^−1^ NMPs are displayed in Figure 2.

## 4. Conclusions

Sheep’s milk is a widely consumed food due to its extraordinary nutritional value, with a high content of protein and essential nutrients, such as nucleotides. However, it can also contain residues related to the use of veterinary drugs, whose maximum residue level in sheep’s milk is regulated by the European Union. For this reason, the development of analytical methodologies that allow the simultaneous determination of contaminants and high-value-added compounds is required. To date, because of the differences in the physicochemical properties between both groups of compounds, different chromatographic modes for their separation, such as RPLC and HILIC, have been used. To the best of our knowledge, this study proposes for the first time the use of 2D-LC in heart-cutting mode for the simultaneous quantification of NMPs (high polarity) and veterinary residues (medium and low polarity) in sheep’s milk. Thus, the separation of veterinary residues has been based on hydrophobic interactions in reversed phase, while the separation of NMPs has been based on ionic interactions. The analytical characteristics of the method in terms of recovery, selectivity, sensitivity, and precision comply with the requirements of the European Commission Decision 2002/657/EC. The proposed method is simple, fast, cost-effective, ecofriendly, allows the analysis of sheep’s milk samples for the quantification of both groups of compounds in less than an hour, and requires instrumentation common in food control and safety laboratories, which would facilitate its implementation as a routine methodology. As limitations and directions for further research for the methodology here proposed, it should be noted that the implementation of mass spectrometry detection would allow to achieve better sensitivity and a better compound confirmation.

## Figures and Tables

**Table 1:** Chromatographic factors for the veterinary drugs separation were obtained for different elution programs.

Chromatographic Separation	Analyte	^a^ t_r_ (min)	^b^ k	^c^ α
Gradient elution method 1	ANH_2_	3.7	2.4	-
	TMP	5.3	3.8	1.62
	ASO	9.2	7.4	1.93
	CPF	14.7	12.4	1.68
	ASO2	16.0	13.5	1.10
	ERF	16.9	14.4	1.06
Gradient elution method 2	ANH2	6.1	4.5	-
	TMP	7.0	5.4	1.18
	ASO	11.4	9.4	1.63
	CPF	12.4	10.3	1.10
	ASO2	13.5	11.3	1.10
	ERF	14.0	11.7	1.04
Gradient elution method 3	ANH2	8.4	6.6	-
	TMP	9.3	7.5	1.15
	ASO	10.5	8.9	1.20
	CPF	11.4	9.4	1.10
	ASO2	12.5	10.4	1.11
	ERF	15.2	12.8	1.24

^a^ Retention time. ^b^ Capacity Factor. ^c^ Separation factor.

**Table 2:** Chromatographic factors for the monophosphate nucleotides separation obtained for isocratic mode and a mixture of acetonitrile:water (10:90%, *v*:*v*) containing sodium acetate (60 mM), 4.5 pH.

Chromatographic Separation	Analyte	^a^ t_r_ (min)	^b^ k	^c^ α
Isocratic elution method	CMP	11.2	9.2	
	UMP	13.0	10.8	1.5
	GMP	14.8	12.5	1.4
	IMP	16.5	14.0	1.2
	AMP	17.7	15.1	1.2

^a^ Retention time. ^b^ Capacity Factor. ^c^ Separation factor.

**Table 3:** Analytical figures of merit of the proposed method and maximum residue levels (MRLs).

Chromatographic Separation	Analyte	^a^ Linear Range (mg L^−1^)	Slope (L mg^1^)	^b^ r	^c^ MQL (mg L^−1^)	^d^ MRL (mg L^−1^)	Recovery (%) ± ^g^ SD			Repeatability (^h^ CV%)			Reproducibility (^h^ CV%)		
							^i^ L1	^j^ L2	^k^ L3	^i^ L1	^j^ L2	^k^ L3	^i^ L1	^j^ L2	^k^ L3
1D	ANH_2_	0.01–0.5	0.2759	0.9982	0.01	^e^ 0.1	70 ± 4	78 ± 4	80 ± 4	6	5	5	13	8	7
	TMP	0.025–0.5	0.1460	0.9957	0.025	0.05	84 ± 3	90 ± 4	95 ± 1	4	4	1	4	5	2
	ASO	0.01–0.5	0.3513	0.9995	0.01	^e^ 0.1	95 ± 5	119 ± 2	108 ± 2	5	2	2	6	4	3
	CPF	0.025–0.5	0.6130	0.9879	0.025	^f^ 0.1	106 ± 10	120 ± 5	114 ± 7	9	4	6	11	7	6
	ASO_2_	0.025–0.5	0.5744	0.9789	0.025	^e^ 0.1	108 ± 4	118 ± 3	109 ± 3	3	3	3	4	4	3
	ERF	0.025–0.5	0.3569	0.9997	0.025	^f^ 0.1	84 ± 4	86 ± 3	82 ± 2	5	4	3	7	5	4
2D	CMP	0.25–100	13.893	0.9984	0.25	-	82 ± 10	83 ± 4	93 ± 6	12	5	6	14	8	8
	UMP	0.5–100	11.617	0.9838	0.5	-	80 ± 11	91 ± 6	117 ± 10	14	7	8	15	10	9
	GMP	0.75–100	6.542	0.9967	0.75	-	81 ± 8	89 ± 3	90 ± 4	10	4	4	12	7	6
	IMP	0.5–100	13.964	0.9983	0.5	-	85 ± 2	83 ± 3	85 ± 2	3	4	3	4	5	3
	AMP	0.25–100	19.880	0.9997	0.25	-	80 ± 5	119 ± 12	118 ± 4	7	10	4	10	10	7

^a^ Instrumental quantitation limit calculated by using a signal-to-noise ratio of 10. ^b^ Correlation coefficient. ^c^ Method quantification limits. ^d^ Maximum residue limits. ^e^ Sum of albendazole sulphoxide, albendazole sulphone, and albendazole 2-amino sulphone, expressed as albendazole. ^f^ Sum of enrofloxacin and ciprofloxacin. ^g^ Standard deviation. ^h^ Coefficient of variation. ^i^ Level 1 of fortification: 0.5 times MRLs of veterinary residues and 5 mg L^−1^ MNPs. ^j^ Level 2 of fortification: 1 times MRLs of veterinary residues and 10 mg L^−1^ MNPs. ^k^ Level 3 of fortification: 1.5 times MRLs of veterinary residues and 15 mg L^−1^ MNPs.

**Table 4:** Mean percent recoveries and standard deviations obtained in the determination of veterinary residues and NMPs in sheep milk from different brands.

Chromatographic Separation	Analyte	Sample 1		Sample 2		Sample 3	
		^a^ R (%) ± ^b^ SD	^c^ Conc. (mg L^−1^) ± ^b^ SD	^a^ R (%) ± ^b^ SD	^c^ Conc. (mg L^−1^) ± ^b^ SD	^a^ R (%) ± ^b^ SD	^c^ Conc. (mg L^−1^) ± ^b^ SD
1D	ANH_2_	68 ± 10	0.675 ± 0.015	78 ± 12	0.578 ± 0.022	68 ± 3	nd
	TMP	85 ± 13	0.085 ± 0.005	82 ± 5	0.015 ± 0.001	87 ± 12	nd
	ASO	122 ± 2	nd	123 ± 3	nd	122 ± 4	nd
	CPF	123 ± 3	nd	116 ± 4	nd	120 ± 7	nd
	ASO_2_	120 ± 6	nd	113 ± 2	nd	103 ± 4	nd
	ERF	79 ±2	nd	84 ± 9	nd	80 ± 9	nd
2D	CMP	88 ± 3	nd	93 ± 11	nd	96 ± 2	nd
	UMP	122 ± 6	0.7 ± 0.1	119 ± 7	<MQL	115 ± 2	1.6 ± 0.2
	GMP	80 ± 2	nd	88 ± 8	nd	91 ± 1	nd
	IMP	81 ± 3	nd	83 ± 7	nd	84 ± 1	nd
	AMP	84 ± 2	16 ± 1	84 ± 1	15.2 ± 0.3	86 ± 2	15.4 ± 0.8

^a^ Recovery calculated for sample fortified at the MRLs of veterinary drug residues and NMPs at 15 mg L^−1^. ^b^ Standard deviation. ^c^ Calculated concentration of the analyte for a non-fortified sample.

## Data Availability

The original contributions presented in the study are included in the article/Supplementary Material, further inquiries can be directed to the corresponding author.

## Supplementary Materials

The following supporting information can be downloaded at: https://www.mdpi.com/article/10.3390/foods13060872/s1; Table S1: Chromatographic methods tested. Solvent A: formic acid (pH: 3), Solvent B: acetonitrile. Injection volume: 50 µL and flow rate: 0.2 mL min^−1^; Table S2: Chemical structure, ionization constant, octanol–water partition coefficient and number of proton donors and acceptors for the veterinary drugs and monophosphate nucleotides; Table S3: Chromatographic methods tested. Solvent A: H_2_O/Acetonitrile (98/2%, *v/v*) + 30 mM sodium acetate, pH: 4.5. Solvent B: H_2_O/Acetonitrile (98/10%, *v/v*) + 60 mM sodium acetate, pH: 4.5. Volume of elution fraction from 1D: 40 µL and flow rate: 0.2 mL min^−1^.
