# Supplementary material for: Heart-Cutting Bidimensional Liquid Chromatography for the Simultaneous Analysis of Veterinary Drugs Residues and Nucleotide Monophosphates in Sheep’s Milk
Source: Foods. 2024 Mar 13;13(6):872. doi: 10.3390/foods13060872 (PMC10969737; doi:10.3390/foods13060872)
Supplement: Supplementary file 1 [file foods-13-00872-s001.zip › foods-2888476-supplementary.pdf]

Table S1

Chromatographic methods tested. Solvent A: formic acid (pH: 3), Solvent B: acetonitrile. Injection volume: 50 µL and flow rate: 0.2 mL min^{-1}.

	Time (min)	Solvent B (%)
**Gradient elution method 1**	0	20
	7.5	20
	8	30
	18	30
	19	90
	26	90
	30	20
**Gradient elution method 2**	0	10
	5	20
	9	20
	12	90
	15	90
	15.5	10
	20	10
**Gradient elution method 3**	0	10
	5	15
	5.5	20
	8	20
	10	90
	12	90
	12.5	10
	20	10

Table S2

Chemical structure, ionization constant, octanol–water partition coefficient and number of proton donors and acceptors for the veterinary drugs and monophosphate nucleotides.

Compound name	Structure	MW[a] (g/mol)[b]	pKa[b]	Log P[b]	Hydrogen Donors/Acceptors[c]
Trimethoprim (TMP)	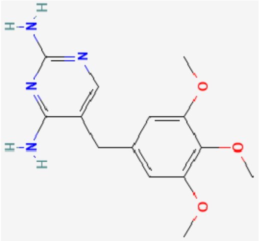	290.32	7.04	0.594	2/7
Albendazole 2-amino sulfone (ANH_2)	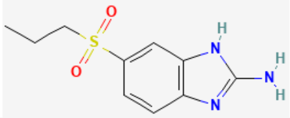	239.30	9.90	0.858	2/4
Enrofloxacin (ERF)	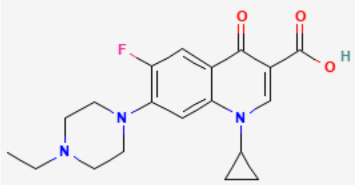	359.4	6.43	2.306	1/7
Ciprofloxacin (CPF)	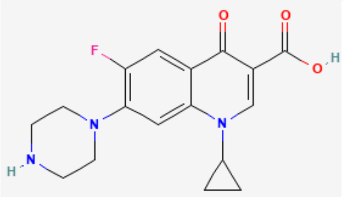	331.34	6.43	1.625	2/7
Albendazole sulfoxide (ASO)	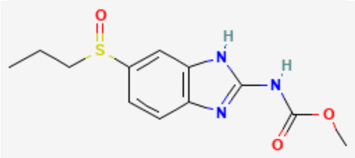	281.33	10.05	0.676	2/5

Albendazole sulfone (ASO_2)	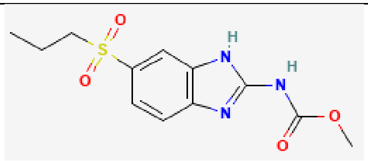	297.33	10.11	1.227	2/5
Cytidine 5′-monophosphate (CMP)[c]	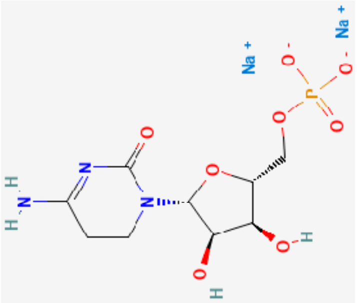	367.16	1.86	-5.134	3/8
Adenosine 5′-monophosphate (AMP)[c]	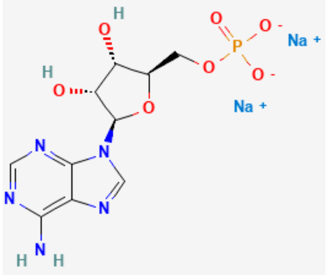	391.19	1.86	-3.190	3/11
Inosine 5′-monophosphate (IMP)[c]	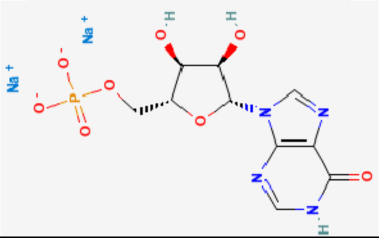	392.17	1.86	-4.227	3/10

Uridine 5′-monophosphate (UMP)	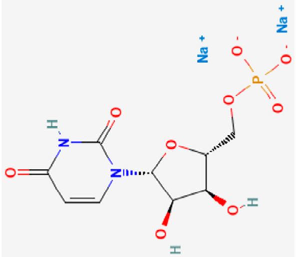	368.14	1.86	-3.452	3/9
Guanosine 5′-monophosphate (GMP)	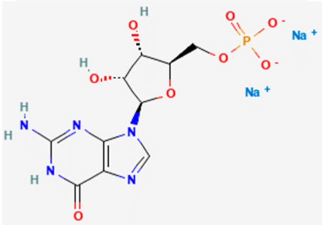	407.18	1.86	-4.067	4/10

Mw: molecular weight. [b]: Calculated using Advanced Chemistry Development (ACD/Labs) Software V11.02 (© 1994-2023 ACD/Labs). [c]: Data obtained from PubChem (https://pubchem.ncbi.nlm.nih.gov/)

Table S3

Chromatographic methods tested. Solvent A: H_2O/Acetonitrile (98/2 %, v/v) + 30mM sodium acetate, pH: 4.5. Solvent B: H_2O/Acetonitrile (98/10 %, v/v) + 60mM sodium acetate, pH: 4.5. Volume of elution fraction from 1D: 40 µL and flow rate: 0.2 mL min^{-1}.

	**Time (min)**	**Solvent B (%)**
**Gradient elution method 1**	0	0
	12	100
	16	100
	20	0
**Gradient elution method 2**	0	95
	15	100
	20	100
	20.5	95
	30	95
**Isocratic elution method 3**	0	100
	20	100
